# Genome-Wide Association Study for Wool Production Traits in a Chinese Merino Sheep Population

**DOI:** 10.1371/journal.pone.0107101

**Published:** 2014-09-30

**Authors:** Zhipeng Wang, Hui Zhang, Hua Yang, Shouzhi Wang, Enguang Rong, Wenyu Pei, Hui Li, Ning Wang

**Affiliations:** 1 Key Laboratory of Animal Genetics, Breeding and Reproduction, Education Department of Heilongjiang province, Harbin, P. R. China; 2 College of Animal Science and Technology, Northeast Agricultural University, Harbin, P. R. China; 3 Institute of Animal Husbandry and Veterinary, Xinjiang Academy of Agricultural and Reclamation Science, Shihezi, P.R. China; China Agricultrual University, China

## Abstract

Genome-wide association studies (GWAS) provide a powerful approach for identifying quantitative trait loci without prior knowledge of location or function. To identify loci associated with wool production traits, we performed a genome-wide association study on a total of 765 Chinese Merino sheep (JunKen type) genotyped with 50 K single nucleotide polymorphisms (SNPs). In the present study, five wool production traits were examined: fiber diameter, fiber diameter coefficient of variation, fineness dispersion, staple length and crimp. We detected 28 genome-wide significant SNPs for fiber diameter, fiber diameter coefficient of variation, fineness dispersion, and crimp trait in the Chinese Merino sheep. About 43% of the significant SNP markers were located within known or predicted genes, including *YWHAZ, KRTCAP3, TSPEAR, PIK3R4, KIF16B, PTPN3, GPRC5A, DDX47, TCF9, TPTE2, EPHA5* and *NBEA* genes. Our results not only confirm the results of previous reports, but also provide a suite of novel SNP markers and candidate genes associated with wool traits. Our findings will be useful for exploring the genetic control of wool traits in sheep.

## Introduction

Sheep (*Ovis aries*) are one of the earliest domesticated animals. Sheep provide humans with a source of meat, milk, wool, and skins, and play a vital role in the global agricultural economy. The Merino sheep is an economically influential breed of sheep prized for its wool. Merino wool is regarded as the finest and softest wool of any sheep. Wool quality traits, such as fiber diameter, length and strength, are important goals in Merino breeding programs. Most countries have high-quality performance measurement programs and well-developed tools for index selection using best linear unbiased prediction (BLUP). However, in the field of sheep breeding for high wool quality, it is difficult to accurately evaluate the genetic component for wool quality traits since the phenotypes of these traits are difficult to be measured.

Over recent decades, advances in DNA-based marker technology have made it possible to identify genomic regions or quantitative trait loci (QTLs) underlying complex traits, such as fiber diameter, in Merino sheep. Instead of traditional animal breeding programs solely relying on phenotype and pedigree information, the incorporation of detected QTLs into genetic evaluation has the potential to enhance selection accuracy, thereby expediting the genetic improvement of animal productivity. A number of papers have been published concerning the detection of QTLs for wool traits. To date, 31 QTLs for wool traits have been reported via genome scan based on marker-QTL linkage analyses (http://cn.animalgenome.org/cgi-bin/QTLdb/OA/index, Aug, 2013) [1]. The limitations of QTL mapping using linkage analysis (LA) and/or linkage disequilibrium (LD), based on panels of low to moderate-density markers, have been well documented [2], [3]. Only a few major genes, e.g., the *KRTAP6* gene (keratin-associated protein 6) [4], the *PROP1* gene (PROP paired-like homeobox 1) [5] and *ADRB3* (beta3-adrenergic receptor) [6], have been associated with wool traits using QTL linkage analyses or candidate gene studies.

With the advent of genome-wide panels of single nucleotide polymorphisms (SNPs), it has become possible to identify and localize QTLs for complex traits in many livestock species [7], including cattle [8]–[12], Swine [13]–[15], chicken [16]–[18], sheep [19]–[22], horse [23], [24], dog [25], [26], and also in humans [27]–[35] using the approach of a genome-wide association study (GWAS). Compared with traditional QTL mapping strategies, a GWAS has major advantages both in its power to detect causal variants with modest effects and in defining narrower genomic regions harboring causal variants for economically important traits. GWASs have been widely accepted as a primary approach for gene identification and have achieved some success in identifying genes conferring modest disease risks in humans [27]–[35].

To date, only a small number of GWASs in sheep have been conducted because of limited information available for the sheep genome. These studies mainly focused on diseases [19], [20], morphology [21], milk production [22] and meat production traits [36]. A group of consecutive SNPs was found to be associated with *Chondrodysplasia* (a condition in which the legs are malformed) [19]. A mutation of the *DMP1* gene was identified to be associated with inherited rickets of Corriedale sheep by a GWAS [20].

Johnston et al.. [21] determined the main candidate gene for sheep horn size and horn-type as *RXFP2*. García-Gámez et al.. [22] identified the *LALBA* gene influencing milk protein percentage in dairy sheep. Zhang et al.. [36] found that five genes are likely to be the most crucial candidate genes associated with post-weaning weight gain, including *MEF2B, RFXANK, CAMKMT, TRHDE*, and *RIPK2*. Nevertheless, no GWAS for sheep wool traits has been performed.

The main objective of this study was to detect significant SNP loci for wool traits on a genome-wide scale using the Illumina sheep SNP50 BeadChip, and to explore potential causal genetic variants and major candidate genes for wool traits.

## Materials and Methods

### Animal population

All animal work was conducted according to the guidelines for the care and use of experimental animals established by the Ministry of Science and Technology of the People's Republic of China (Approval number: 2006–398) and was approved by the Laboratory Animal Management Committee of Northeast Agricultural University.

A total of 765 Chinese Merino sheep (JunKen type) used in this study came from the Xinjiang Academy of Agricultural and Reclamation Science. These sheep were from six strains of Chinese Merino sheep (strain A (n = 159), strain B (n = 103), strain U (n = 35), strain D (n = 145), strain DR (n = 135) and strain X (n = 188)). All of these sheep were located in the same farm. We measured and recorded five wool production traits: fiber diameter, fiber diameter coefficient of variation, fineness dispersion, staple length and crimp. The descriptive statistics of the phenotypic measurements of the sheep used for the GWAS are given in Table 1.

**Table 1:** Descriptive statistics of phenotype values of wool traits for sheep population.

Traits	Mean	Standard deviation	Minimum	Maximum	Number
Fiber Diameter	20.42	1.93	15.46	28.33	765
Fiber Diameter Coefficient of variation	4.10	0.66	2.62	6.79	765
Fineness Dispersion	20.11	2.72	7.70	36.50	765
Staple Length	9.26	1.27	5.00	13.00	765
Crimp	12.48	2.48	6.00	19.00	764

### SNP genotyping and selection

Genomic DNA extraction from sheep ear tissue was performed by the phenol-chloroform method. The DNA was stored at −20°C. Genotyping of the Sheep 50 K SNP chips from Illumina Inc. was performed by DNA LandMarks Inc. (Quebec, Canada) using 75 µL of approximately 50 ng/µL genomic DNA. BeadStudio software with the genotyping module (Illumina, 2006) was used to determine the genotypes of the individuals used in the current study.

Following quality control, 47,286 SNPs with minor allele frequencies (MAF) of 5% or greater and call rates of 95% or greater were selected for use in this study. These SNPs were distributed across 26 autosomes and the X chromosome, with the number of SNPs per chromosome ranging from 640 to 5161, and with a mean distance between adjacent SNPs ranging from 53.4 to 100.7 kb (for details see Table S1). Individuals with 5% or more missing SNP genotypes were excluded.

### Statistical analyses

#### SNP association analysis

In this study, the statistical model was Y = μ+ Line + Year+ SNP + Animal +e, where Y was the phenotype value, Line was the strains effect, Year was ages of the animals when they were phenotyped on the traits studied, SNP was the SNP marker effect, Animal was a random animal effect to account for individual correlation, and e was the residual effect. Approximately 50% of the pedigree information was missing in this study population. So a genomic relationship inferred from the 50K SNP data can be inferred and reflect the similarity of paired individuals. Using the genome association and prediction integrated tool (GAPIT) [37] in R v3.0.3, we performed single marker mixed-model GWAS for each wool trait. The R package GAPIT was used to generate the matrix using the EMMA algorithm [41]. We use SNPEVG tool [42] to show the quantile-quantile plot and Manhattan plots for each GWAS result.

#### Statistical inference

The Bonferroni method was used to adjust for multiple testing from the number of SNP loci detected. We declared a significant SNP at the genome-wide significance level if the raw *P*-value was <0.05/*N*, here *N* is the number of SNP loci tested in the analyses. In this study, for each trait, the threshold *P*-value for declaring genome-wide significance was (0.05)/47,286 = 1.06×10^−6^ = 10^−5.97^ (47,286 SNP markers).

### Identification of candidate genes

Sheep transcripts and annotations were downloaded from the sheep UCSC database (build 3.0). In addition, the human-sheep comparative map and the syntenic regions annotations were also downloaded from the UCSC database.

## Results and Discussion

**Figure 1:**
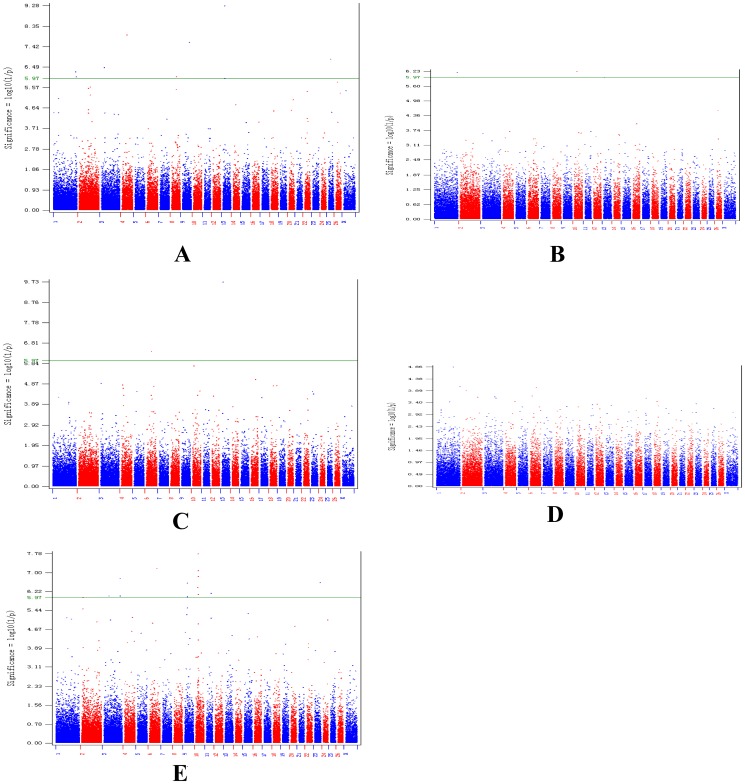
Manhattan plot of genome-wide association analysis for five wool production Traits. The horizontal solid line declares genome-wise 5% significance with a p-value threshold of 1.06×10^−6^. Fig. 1-A, 1-B, 1-C, 1-D and 1-E refer to plots for fiber diameter, fiber diameter coefficient of variation, fineness dispersion, crimp and staple length trait, respectively. Red triangle refers to significant SNPs plots.

**Figure 2:**
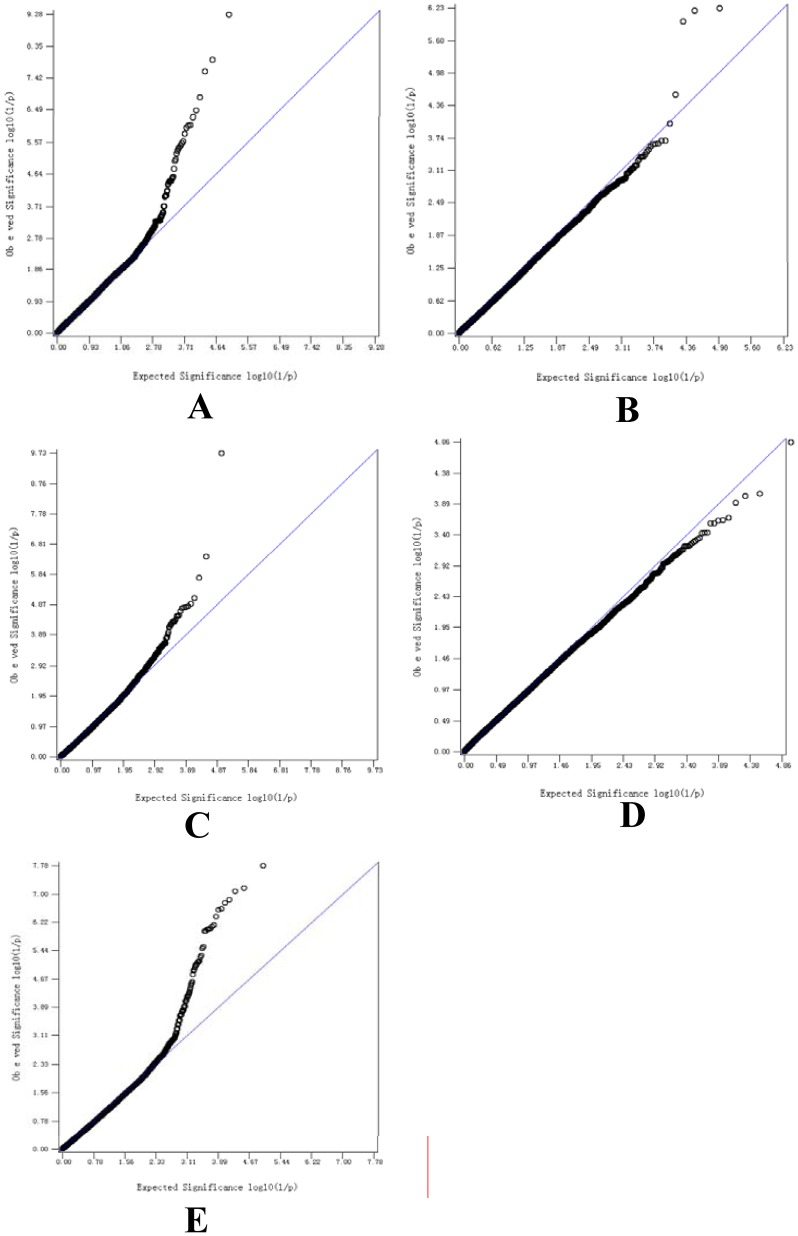
Quantile-quantile (Q-Q) plot of genome-wide association analysis for five wool production Traits. Fig. 2-A, 2-B, 2-C, 2-D and 2-E refer to plots for fiber diameter, fiber diameter coefficient of variation, fineness dispersion, crimp and staple length trait, respectively.

The global view of P-values (in terms of - log(P-value)) for all SNP markers of each trait are represented by a Manhattan plot shown in Fig. 1. And the quantile-quantile plot for each GWAS result show in Fig. 2. The numbers of genome-wide significant SNPs detected for the four traits (fiber diameter, fiber diameter coefficient of variation, fineness dispersion, crimp) were 9, 3, 2 and 15, respectively, and the details of these significant SNPs, including their positions in the genome, the nearest known genes and the raw P-values, are given in Table 2. On the other hand, there were no significant SNPs for staple length traits.

**Table 2:** Genome-wise significant (p<0.05) SNPs for wool production traits.

Traits	SNP Name	OAR	Position(bp)	Nearest gene		Raw P value
				Name	Distance (bp)	
Fiber Diameter	s73369.1	1	262,567,035	*TSPEAR*	within	5.18E-07
Fiber Diameter	s68599.1	1	269,615,805	*PIK3R4*	within	8.90E-07
Fiber Diameter	s14929.1	3	34,364,400	*KRTCAP3*	within	3.36E-07
Fiber Diameter	OAR4_55981443.1	4	52,838,065	*TFEC*	396.1 kb	1.11E-08
Fiber Diameter	OAR8_53159504.1	8	49,600,831	*SLC35A1*	14.4 kb	8.64E-07
Fiber Diameter	OAR9_80743202.1	9	76,045,867	*YWHAZ*	within	2.43E-08
Fiber Diameter	OAR13_19523580.1	13	17,163,024	*CCNY*	6.6 kb	5.27E-10
Fiber Diameter	OAR13_19577291.1	13	17,218,383	*CREM*	19.3 kb	1.03E-06
Fiber Diameter	OAR25_21582203.1	25	20,863,365	*CFDP2*	136.6 kb	1.39E-07
fiber diameter coefficient of variation	OAR1_293501261.1	1	270,981,547	*Mir563*	141 bp	6.57E-07
fiber diameter coefficient of variation	s41906.1	10	30,072,709	*HSPA1*	7.9 kb	5.95E-07
fiber diameter coefficient of variation	s40917.1	13	9,809,552	*KIF16B*	within	1.06E-6
Fineness dispersion	OAR6_63321833.1	6	57,451,934	*TBC1D1*	52.2 kb	3.90E-07
Fineness dispersion	s40917.1	13	9,809,552	*KIF16B*	within	1.86E-10
Crimp	s54656.1	2	13,824,172	*PTPN3*	within	1.04E-06
Crimp	OAR3_66317995.1	3	62,704,565	*TCF9*	within	9.14E-07
Crimp	s30057.1	3	201,873,340	*GPRC5A*	within	1.75E-07
Crimp	OAR3_217297404.1	3	201,904,310	*DDX47*	within	9.31E-07
Crimp	OAR6_88473191.1	6	81,031,420	*EPHA5*	within	6.83E-08
Crimp	s22461.1	9	29,885,716	*ZHX2*	162.6 kb	2.70E-07
Crimp	OAR9_32239874.1	9	30,811,476	*HAS2*	133.8 kb	1.05E-06
Crimp	OAR9_32280523.1	9	30,852,525	*HAS2*	92.7 kb	9.30E-07
Crimp	s46011.1	10	22,028,163	*TPTE2*	within	4.14E-07
Crimp	OAR10_26266695.1	10	26,350,351	*NBEA*	within	8.35E-08
Crimp	OAR10_26652355.1	10	26,716,452	*NBEA*	123.9 kb	1.67E-08
Crimp	OAR10_26950904.1	10	26,990,970	*SLC25A5*	379.1 kb	1.43E-07
Crimp	OAR10_27067374.1	10	27,100,665	*SLC25A5*	269.4 kb	7.87E-07
Crimp	OAR11_48526914.1	11	45,603,254	*CDC27*	26.2 kb	7.12E-07
Crimp	OAR23_58971409.1	23	55,437,569	*TCF4*	258.4 kb	2.54E-07

### Fiber diameter

As shown in Table 2, there are 9 significant SNPs for fiber diameter, and they were distributed on seven autosomes. These SNPs were distributed unevenly among the chromosomes, and there were 2, 1, 1, 1, 1, 2, 1 significant SNP markers associated with the fiber diameter trait on OAR1, OAR3, OAR4, OAR8, OAR9, OAR13 and OAR25, respectively. Furthermore, two significant SNPs on OAR13 concentrated in a region of 60 kb (17.16–17.22 Mb).

Four of the 9 significant SNP markers were located within known or predicted genes including the *TSPEAR* (Thrombospondin-type laminin G domain and EAR repeat), *PIK3R4* (phosphoinositide-3-kinase, regulatory subunit 4), *KRTCAP3* (Keratinocyte-associated protein 3) and *YWHAZ* (of the 14-3-3 family of proteins) genes. The others were located 6.6 to 396.1 kb away from the nearest known gene.

The *KRTCAP3* gene is expressed in skin keratinocytes [40]. Keratinocytes are the most common type of skin cells. They make keratin, a protein that provides strength to skin, hair and nails. In this study, marker s14929.1 on chromosome 3 was located within the *KRTCAP3* gene.

The *YWHAZ* gene product belongs to the 14-3-3 family of proteins, which mediate signal transduction by binding to phosphoserine-containing proteins. The encoded protein interacts with the IRS1 protein, suggesting a role in regulating insulin sensitivity. The human *YWHAZ* gene, located on chromosome 8q22.3, is expressed in HNSCC (head and neck squamous cell carcinomas) cases. The *YWHAZ* mRNA is frequently upregulated in tumor tissues. Furthermore, the *YWHAZ* gene-specific RNAi significantly suppressed the growth rate of HNSCC cell lines, and overexpression of *YWHAZ* in HaCaT immortalized human skin keratinocytes promoted overgrowth, as well as morphological changes [38]. Reduced levels of *YWHAZ* increased the proportion of cells in G1/G0-phase, and decreased the proportion in S-phase and the rate of DNA synthesis. Consequently, Lin et al.. suggested that *YWHAZ* is a candidate proto-oncogene [38]. In this study, the marker OAR9_80743202.1 on chromosome 9 had the most significant association with the fiber diameter trait. This marker was located within the *YWHAZ* gene.

The *CCNY* gene controls cell division cycles and regulates cyclin-dependent kinases. Franke et al.. found that SNP marker rs3936503 in the *CCNY* gene on chromosome 10p11.2 was related to Crohn's disease, based on a sample of 1850 German Crohn's disease patients and 1,817 controls [39]. Crohn's disease is a type of inflammatory bowel disease that causes abdominal pain, diarrhea, vomiting or complications outside the gastrointestinal tract, such as skin rashes and arthritis. In this study, the SNPs (OAR13_19523580.1) on the ORA13, were located near the *CCNY* gene from 6.6 kb.

The functions of all of the above genes are directly or indirectly related to skin development. Hair follicles are skin appendages and produce hair; therefore, we hypothesize that these genes control hair follicle development and fiber diameter trait.

On chromosomes OAR1, OAR5, OAR6, OAR13 and OAR25, 7 QTLs were reported to be related to fiber diameter [1]. Of these QTLs, one QTL was located on OAR1, ranging from 277.8 to 293.1 cM [44]. In present study, an SNP marker (s73369.1) was located at 262.6 Mb on OAR1. Vitezica et al. (2007) identified one QTL on OAR13 at 94.1 cM, controlling fiber diameter trait in INRA 401 sheep [45]. In this study, we detected one SNP located in the 17.1 Mb regions on OAR13. Allain et al. (2006) identified one QTL on OAR25 at 6.4 cM, controlling fiber diameter trait in a sheep backcross SARDA and LACAUNE resource population [46]. Bidinost et al. (2006) identified one QTL on OAR25 at 52.6 cM, controlling fiber diameter trait in Merino sheep [47]. Ponz et al. (2001) identified one QTL on OAR25 at 68.3 cM, controlling fiber diameter trait in INRA 401 sheep [48]. In this study, we detected one SNP located in the 20.9 Mb regions on OAR25. Moreover there were one QTLs detected on OAR5 and OAR6, respectively [5], [48]. However, in this study, there were no significant SNP markers identified on these chromosomes.

### Fiber diameter coefficient of variation

As shown in Table 2, three significant SNPs were distributed onOAR1, OAR10 and OAR13. Only one significant SNP was located *KIF16B* gene; the others were not located within known or predicted genes. SNP marker OAR1_293501261.1 on OAR1 is 141 bp downstream from the 3′ end of the closest gene, *Mir563*. Target gene search using TargetScan [43] showed *Mir563* has 36 target genes in human, including *FBN1* (fibrillin 1), *KIAA0831* (for details see *Mir563* target genes see Table S2).

There were one, one, two and three QTLs on OAR4, OAR7, OAR11, OAR25, respectively, related to fiber diameter coefficient of variation trait [44], [46]–[48]. However, in this study no significant SNPs markers were located on these chromosomes.

### Fineness dispersion

As shown in Table 2, only two significant SNPs (OAR6_63321833.1 and s40917.1) were detected. These SNPs were located at OAR6 and OAR13, respectively. In addition, SNP markers s40917.1 was also significantly associated with fiber diameter coefficient of variation. That SNP marker was located within *KIF16B* gene (KIF16B kinesin family member 16B).

### Staple length

No significant SNPs were detected for staple length trait. However, several papers have been published concerning detection of QTLs for the staple length trait, and seven were detected on chromosomes 3, 7, 14, 15, 18 and 25. Ponz et al.. typed 40 microsatellites covering 20 autosomes on the synthetic breed INRA401 population, which is a composite Romanov (prolific breed) and Berrichon du Cher (meat breed), and detected three QTLs on chromosomes 3, 7 and 25 related to staple length [48]. Allain et al.. suggested four putative QTLs for staple length on chromosomes 14, 15, 18 and 25 [46]. However, in the present study, no significant SNP markers were associated with these QTLs for staple length.

### Crimp

Fifteen significant SNPs were detected (for details, see Table 2). These SNPs were distributed on OAR2, OAR3, OAR6, OAR9, OAR10, OAR11 and OAR23. The four significant SNPs concentrated in a region of 0.7 Mb (26.4–27.1 Mb) on chromosome 10. In addition, seven significant SNPs were located within known or predicted genes, including *PTPN3* (Tyrosine-protein phosphatase non-receptor type 3), *TCF9* (GC-rich sequence DNA-binding factor 2), *GPRC5A* (G protein-coupled receptor, family C, group 5, member A), *DDX47* (DEAD (Asp-Glu-Ala-Asp) box polypeptide 47), *EPHA5* (EPH receptor A5), *TPTE2* (transmembrane phosphoinositide 3-phosphatase and tensin homolog) and *NBEA* (neurobeachin), and the other SNPs were located 26.2 kb to 379.1 kb away from the nearest known genes.

The *GPRC5A* gene is located on OAR3 from 201.83 Mb to 201.88 Mb. This gene encodes a member of the type 3 G protein-coupling receptor family, characterized by a characteristic 7-transmembrane domain motif. The encoded protein may be involved in the interaction between retinoid acid and G protein signaling pathways. This gene may be involved in modulating differentiation and maintaining homeostasis of epithelial cells [49]. In this study, markers s30057.1 within *GPRC5A* gene were significantly related to the crimp trait.

The *EPHA5* gene, located on OAR6 from 80.71 Mb to 81.09 Mb, encodes a protein that belongs to the ephrin receptor subfamily of the protein-tyrosine kinase family. One SNP marker OAR5_43897574.1, which fall within this *EPHA5* gene, was most significantly associated with the crimp trait. This SNP is 33.1 kb downstream from the 3′ end of the closest gene, *SH3BP5L* (SH3 domain-binding protein 5-like). The encoded protein SH3BP5L interacts with YWHAZ protein [50]. As mentioned before, the *YWHAZ* gene harbors the marker OAR9_80743202.1, which was significantly associated with sheep fiber diameter in this study.

As mentioned above, none of these genes plays an obvious role in the crimp trait. But, the functions of all of the above genes are directly or indirectly related to epithelial cells or skin development. So we presume these genes may be involved in determining the crimp trait. A future study will investigate the biological functions of these genes.

## Conclusions

In the present study, we detected 28 genome-wide significant SNPs for fiber diameter, fiber diameter coefficient of variation, fineness dispersion and crimp trait in a Chinese Merino sheep (JunKen type) populations. Some significant SNP markers were located within previously reported candidate genes. However, most of the candidate genes and SNP markers, for the first time, were reported as related to wool production traits. Our findings lay the basis for follow-up replication studies, which will reveal the causal mutations underlying wool production traits in Merino sheep.

## Supporting Information

Table S1 **Distributions of SNPs after quality control and the average distances between adjacent SNPs on each chromosome.**
(DOC)

Table S2 **All targeted genes by Mir563 in human.**
(DOC)
