# Supplementary material for: Genome-Wide Association Study for Wool Production Traits in a Chinese Merino Sheep Population
Source: PLoS One. 2014 Sep 30;9(9):e107101. doi: 10.1371/journal.pone.0107101 (PMC4182092; doi:10.1371/journal.pone.0107101)
Supplement: Table S1 — Distributions of SNPs after quality control and the average distances between adjacent SNPs on each chromosome. (DOC) [file pone.0107101.s001.doc]

Table S1. Distributions of SNPs after quality control and the average distances between adjacent SNPs on each chromosome.

| OAR | No. SNPs | Length including gaps (kb) | Average distance (kb) |
| --- | --- | --- | --- |
| 1 | 5161 | 299,840 | 58.1 |
| 2 | 4842 | 263,237 | 54.4 |
| 3 | 4381 | 242,888 | 55.4 |
| 4 | 2369 | 128,105 | 54.1 |
| 5 | 2093 | 117,186 | 56.0 |
| 6 | 2280 | 129,067 | 56.6 |
| 7 | 1970 | 108,951 | 55.3 |
| 8 | 1835 | 97,956 | 53.4 |
| 9 | 1883 | 100,831 | 53.5 |
| 10 | 1612 | 94,216 | 58.4 |
| 11 | 1021 | 67,138 | 65.8 |
| 12 | 1509 | 86,458 | 57.3 |
| 13 | 1489 | 89,095 | 59.8 |
| 14 | 1025 | 69,343 | 67.7 |
| 15 | 1476 | 90,133 | 61.1 |
| 16 | 1382 | 77,188 | 55.9 |
| 17 | 1257 | 78,643 | 62.6 |
| 18 | 1239 | 72,490 | 58.5 |
| 19 | 1109 | 64,989 | 58.6 |
| 20 | 980 | 55,885 | 57.0 |
| 21 | 790 | 55,550 | 70.3 |
| 22 | 954 | 55,886 | 58.6 |
| 23 | 1006 | 66,771 | 66.4 |
| 24 | 640 | 45,319 | 70.8 |
| 25 | 890 | 48,347 | 54.3 |
| 26 | 811 | 50,101 | 61.8 |
| x | 1282 | 129,136 | 100.7 |
