# Supplementary material for: Genome-Wide Association Study for Wool Production Traits in a Chinese Merino Sheep Population
Source: PLoS One. 2014 Sep 30;9(9):e107101. doi: 10.1371/journal.pone.0107101 (PMC4182092; doi:10.1371/journal.pone.0107101)
Supplement: Table S2 — All targeted genes by Mir563 in human. (DOC) [file pone.0107101.s002.doc]

Table S2. All targeted genes by *Mir563* in human.

| Gene Name | Official Full Name |
| --- | --- |
| *RNF111* | ring finger protein 111 |
| *CALML4* | calmodulin-like 4 |
| *PBRM1* | polybromo 1 |
| *PTCHD1* | patched domain containing 1 |
| *SETBP1* | SET binding protein 1 |
| *PTP4A1* | protein tyrosine phosphatase type IVA, member 1 |
| *WNK4* | WNK lysine deficient protein kinase 4 |
| *UTX* | ubiquitously transcribed tetratricopeptide repeat, X chromosome |
| *N4BP3* | Nedd4 binding protein 3 |
| *HNRNPA2B1* | heterogeneous nuclear ribonucleoprotein A2/B1 |
| *KPNA1* | karyopherin alpha 1 (importin alpha 5) |
| *IL33* | interleukin 33 |
| *SFRS7* | splicing factor, arginine/serine-rich 7, 35kDa |
| *TRIM2* | tripartite motif-containing 2 |
| *NUDT4* | nudix (nucleoside diphosphate linked moiety X)-type motif 4 |
| *MDGA2* | MAM domain containing glycosylphosphatidylinositol anchor 2 |
| *FRAS1* | Fraser syndrome 1 |
| *PDE11A* | phosphodiesterase 11A |
| *SMURF1* | SMAD specific E3 ubiquitin protein ligase 1 |
| *AFF4* | AF4/FMR2 family, member 4 |
| *FBN1* | fibrillin 1 |
| *HN1L* | hematological and neurological expressed 1-like |
| *KCNMA1* | potassium large conductance calcium-activated channel, subfamily M, alpha member 1 |
| *MARCKS* | myristoylated alanine-rich protein kinase C substrate |
| *KIAA0831* | KIAA0831 |
| *GPAM* | glycerol-3-phosphate acyltransferase, mitochondrial |
| *CD83* | CD83 molecule |
| *NFAT5* | nuclear factor of activated T-cells 5, tonicity-responsive |
| *CLCN5* | chloride channel 5 (nephrolithiasis 2, X-linked, Dent disease) |
| *STAC* | SH3 and cysteine rich domain |
| *C17ORF39* | chromosome 17 open reading frame 39 |
| *MINK1* | misshapen-like kinase 1 (zebrafish) |
| *METTL8* | methyltransferase like 8 |
| *RPS6KA3* | ribosomal protein S6 kinase, 90kDa, polypeptide 3 |
| *ETNK2* | ethanolamine kinase 2 |
| *DCP1A* | DCP1 decapping enzyme homolog A (S. cerevisiae) |
